# Unmasking Primary Candidemia: Clinical Features and Implications for Management

**DOI:** 10.1111/myc.70228

**Published:** 2026-10-23

**Authors:** Marta Albanell‐Fernández, Andrea Vergara, Marta Bodro, Josep Mensa, Montse Tuset, Sabina Herrera, Álex Soriano

**Affiliations:** ^1^ Pharmacy Service, Division of Medicines, Hospital Clinic of Barcelona University of Barcelona Barcelona Spain; ^2^ Department of Physiological Science, School of Medicine University of Barcelona Barcelona Spain; ^3^ Institut d'Investigacions Biomèdiques August Pi i Sunyer (IDIBAPS) Barcelona Spain; ^4^ Faculty of Medicine University of Barcelona Barcelona Spain; ^5^ Microbiology Department Hospital Clinic of Barcelona, University of Barcelona Barcelona Spain; ^6^ Institute of Global Health (ISGlobal) Barcelona Spain; ^7^ CIBERINFEC, CIBER in Infectious Diseases, ISCIII Madrid Spain; ^8^ Infectious Diseases Department Hospital Clinic of Barcelona, University of Barcelona Barcelona Spain

**Keywords:** candidemia, mortality, *N. glabratus*, translocation

## Abstract

**Background:**

Source identification in candidemia is crucial for management and prognosis; however, a substantial proportion of cases are classified as primary candidemia without an identifiable source.

**Objectives:**

To assess the clinical relevance of primary candidemia and its impact on 7‐day and 30‐day mortality:

**Methods:**

Single‐center retrospective study of adult patients with candidemia from 2011 to 2025 at a tertiary university hospital. Clinical, microbiological, and outcome data were collected, and candidemia sources were classified using predefined clinical and microbiological criteria. Multivariable logistic regression was used to identify factors associated with 7‐ and 30‐day mortality and primary candidemia.

**Results:**

A total of 533 candidemia episodes were analysed; 33.2% were classified as primary candidemia. Overall, 7 and 30‐day mortality were 16.5% and 32.1%. Septic shock (OR 3.6, 95% CI: 2.1–6.3; *p* < 0.001) and primary candidemia (OR 2.2, 95% CI: 1.2–3.8; *p* = 0.011) were independently associated with increased 7‐day mortality. In the cases of primary candidemia, 50.8% of all deaths occurred within the first 5 days. This source was associated with male sex (OR 1.7, 95% CI: 1.1–2.5; *p* = 0.016) and *N. glabratus* infection (OR 2.1, 95% CI: 1.3–3.3; *p* = 0.001). 30‐day mortality was influenced by host‐related factors (liver disease, neutropenia, and haematopoietic stem cell transplantation).

**Conclusion:**

Primary candidemia accounts for a substantial proportion of cases and is associated with high 7‐day mortality. The predominance of *C. albicans* and *N. glabratus* , common gut colonisers, supports gastrointestinal translocation as a potential source. Delayed diagnosis, particularly of *N. glabratus* , highlights the need for appropriate empirical therapy and rapid diagnostic tools.

## 1 Introduction

*Candida* spp. are commensal organisms that colonise mucosal surfaces, particularly the gut and skin of 50%–70% of healthy individuals. In healthy people, the density of *Candida* spp. is limited by microbiome competition; however, factors such as antibiotic exposure, immunosuppression, and corticosteroid use may promote their overgrowth [1, 2, 3]. Disruption of mucosal barriers or increased intestinal permeability can facilitate the translocation of *Candida* spp. into the bloodstream, increasing the risk of candidemia and may lead to deep‐seated secondary infections [2, 3].

Accurate identification of the source of candidemia is a key component of clinical management, as effective source control has consistently been associated with reduced attributable mortality [4, 5]. Sources are traditionally defined based on clinical and microbiological criteria, including catheter‐related, intra‐abdominal, urinary tract, and endocarditis‐associated candidemia, while primary candidemia is defined as bloodstream infection without an identifiable source after appropriate diagnostic evaluation [5, 6, 7, 8].

However, primary candidemia remains a poorly defined and heterogeneous category. It includes patients without an identifiable source who do not meet the criteria for established entities and may also encompass cases in which a catheter‐related source is clinically suspected but not microbiologically confirmed because of negative catheter tip cultures.

Although primary candidemia has been associated with serious outcomes, like intra‐abdominal and endocarditis‐related sources [8, 9], it remains poorly characterised and insufficiently studied. A more precise characterisation is therefore needed to define the clinical profile of these patients better. We aimed to assess the clinical relevance of primary candidemia and its impact on 7‐ and 30‐day mortality.

## 2 Methods

### 2.1 Study Design and Setting

We conducted a retrospective study at an 800‐bed tertiary university hospital. Data on all consecutive BSI diagnosed between 2011 and 2025 were prospectively recorded in a purpose‐designed surveillance database. From this cohort, episodes of *Candida* spp. were identified, and relevant clinical and microbiological variables were analysed.

Collected variables included demographics, hospital ward at the onset of *Candida* BSI, underlying conditions, risk factors for *Candida* BSI, *Candida* species, source of candidemia, absolute neutrophil count, source control, target antifungal therapy, hospital length of stay, and clinical outcomes (7‐day and 30‐day mortality).

Eligible participants were adult inpatients (≥ 18 years) with a confirmed bloodstream infection caused by *Candida* spp. Patients were excluded if key data were missing, including *Candida* spp. identification, relevant clinical variables, or outcome information (e.g., date of death).

The study was approved by the Ethics Committee of the center (HCB/2025/0796). The need for informed consent was waived due to the noninterventional, retrospective nature of the study.

### 2.2 Objectives

Our primary aim was to assess the clinical impact of primary candidemia on 7‐day and 30‐day mortality. Our secondary aim was to identify factors associated with primary candidemia.

### 2.3 Microbiological Procedures

Volumes between 8 and 10 mL of blood samples were inoculated into aerobic and anaerobic bottles and processed using the BACTEC 9240 system from 2011 to 2013, and the BACTEC FX system (Becton–Dickinson, Block Scientific, Bellport, NY, US) from 2013 onward. Incubation was continued for 5 days before bottles were reported as negative, and bottles were loaded into the instrument 24/7. The vials used were resin‐containing BACTEC‐Plus Aerobic/F and BACTEC‐Plus/Anaerobic/F or non‐resin‐containing BACTEC‐Standard 10/Aerobic/F and BACTEC‐Lytic/10/Anaerobic/F. When yeasts were observed on Gram stain, the positive blood culture (BC) was subcultured onto Sabouraud and CHROMagar agar plates (BD BBL Stacker Plates, Heidelberg, Germany). Species identification was performed by MALDI‐TOF mass spectrometry (Bruker) since 2011.

### 2.4 Definitions

An episode of *Candida* BSI was defined as at least one BC positive for *Candida* spp. obtained from a patient with signs and/or symptoms of infection during hospitalisation. The candidemia source was determined based on clinical criteria and the isolation of the same strain from a clinical sample. All candidemia episodes were systematically reviewed by infectious disease specialists, who assessed the most likely source of infection. Catheter‐related candidemia was defined as recovery of the same *Candida* species from peripheral BC and, when performed, from semi‐quantitative catheter‐tip cultures; however, a positive catheter‐tip culture was not required when the catheter was considered the most likely source based on the overall clinical and microbiological assessment. An intra‐abdominal source required clinical and radiological evidence of peritonitis consistent with invasive candidiasis and/or positive cultures with a *Candida* spp. obtained during surgery or by needle aspiration. Urinary tract source was established if the same *Candida* spp. was isolated in the urine culture of a patient with a specific predisposing condition such as urinary catheterisation, urinary tract obstruction, recent urological instrumentation, or surgery. Other sources included endocarditis (definite or probable *Candida* endocarditis according to the clinical, microbiological, and echocardiographic findings) and hepatosplenic (compatible hepatosplenic lesions identified in the context of candidemia). Primary candidemia was defined as an infection with no apparent source that did not match any other source criteria after adequate investigation.

Breakthrough candidemia was defined as candidemia occurring despite active systemic antifungal therapy to which the *Candida* spp. isolate is expected to be susceptible.

Septic shock was defined when the patient required vasopressors due to persistent hypotension despite fluid resuscitation at the time of *Candida* BSI diagnosis [10].

Source control measures included catheter removal, as well as surgical or radiologic procedures to drain abscesses or fluid collections suspected of being the source of *Candida* infection [11]. Early source control was defined as control of the infection source within 24 h of positive BCs [12].

### 2.5 Statistical Analysis

Categorical variables were presented as absolute numbers and percentages, and continuous variables as median and interquartile range (IQR). Associations between categorical variables were assessed using Pearson's chi‐square test or Fisher's exact test, as appropriate.

Univariate and multivariable analyses were performed to identify variables independently associated with primary candidemia, as well as with 7‐day and 30‐day mortality. Variables with a *p*‐value < 0.05 in univariate analyses were included in a multivariate logistic regression model. Model performance and goodness‐of‐fit for the logistic regression models were evaluated using the area under the receiver operating characteristic curve and the Hosmer‐Lemeshow test, respectively.

Analyses were conducted with STATA/SE version 17.0 (StataCorp, College Station, TX, USA). *p*‐values < 0.05 were considered statistically significant.

## 3 Results

During the study period, 544 episodes of *Candida* spp. BSI were identified. Of these, 533 episodes occurring in 518 patients were included due to complete data availability. Overall, 64.4% (*n* = 343) of patients were male, with a median [IQR] age of 65 [55–75] years. At the time of candidemia diagnosis, 42.8% (*n* = 228) were admitted to the intensive care unit. The most frequently isolated species was *Candida albicans* (32.5%, *n* = 173), followed by *Nakaseomyces glabratus* (25.7%, *n* = 137). The most common source of infection was catheter‐related (36.4%, *n* = 194), followed by primary candidemia (33.2%, *n* = 177). 7‐day mortality occurred in 16.5% (*n* = 88) of the cases, while 30‐day mortality was 32.1% (*n* = 171) (Table 1).

**TABLE 1:** Baseline demographic and clinical characteristics of the study cohort.

	*N* = 533 episodes (%)
Demographics	
Age (years), median [IQR]	65 [55–75]
Gender (male), *n* (%)	343 (64.4%)
Hospital location at candidemia onset, *n* (%)	
Intensive care unit	228 (42.8)
General ward	305 (57.2)
Risk factors for candidemia, *n* (%)	
Major surgery within the previous month	173 (32.5)
Total parenteral nutrition	148 (27.8)
Neutropenia, (< 500 cells/μL)	40 (7.5)
Clinical severity at candidemia diagnosis	
Septic shock	161 (30.2)
Mechanical ventilation	167 (31.3)
*Candida* species, *n* (%)	
*C. albicans*	173 (32.5)
*C. parapsilosis*	128 (24.0)
*Nakaseomyces glabratus* ( *C. glabrata* )	137 (25.7)
*C. tropicalis*	52 (9.8)
*Pichia kudriavzevii* ( *C. krusei* )	26 (4.9)
Other *Candida* spp.	17 (3.2)
Breakthrough candidemia, *n* (%)	84 (15.8)
Source of candidemia, *n* (%)	
Catheter‐related	194 (36.4)
Primary candidemia	177 (33.2)
Abdominal	80 (15.0)
Urological	71 (13.3)
Other	11 (2.1)
Invasive devices at candidemia diagnosis	
Urinary catheter	327 (61.4)
Central venous catheter^a^	397 (80.2)
Early source control	176 (33.0)
Antifungal treatment initiated within 24 h of BC collection	183 (34.3)
Initial antifungal treatment, *n* (%)^b^	
Fluconazole	150 (29.7)
Echinocandin	355 (70.3)
Hospital length of stay (days), median [IQR]	37 [20–65]
Death within 7 days from the first positive BC, *n* (%)	88 (16.5)
Death within 30 days from the first positive BC, *n* (%)	171 (32.1)

Abbreviations: BC, blood culture; IQR, interquartile range.

^a^ Among patients with a catheter in place at the time of candidemia diagnosis (*N* = 495).

^b^ Among patients who received antifungal treatment (*N* = 505).

Figure 1A depicts mortality over the first 30 days following candidemia. Nearly half of all deaths (48.8%) occurred within the first 7 days. The lowest 7‐day mortality was observed in catheter‐related candidemia (8.8%), whereas markedly higher rates were seen in other sources (27.3%) and primary candidemia (24.9%).

**FIGURE 1:**
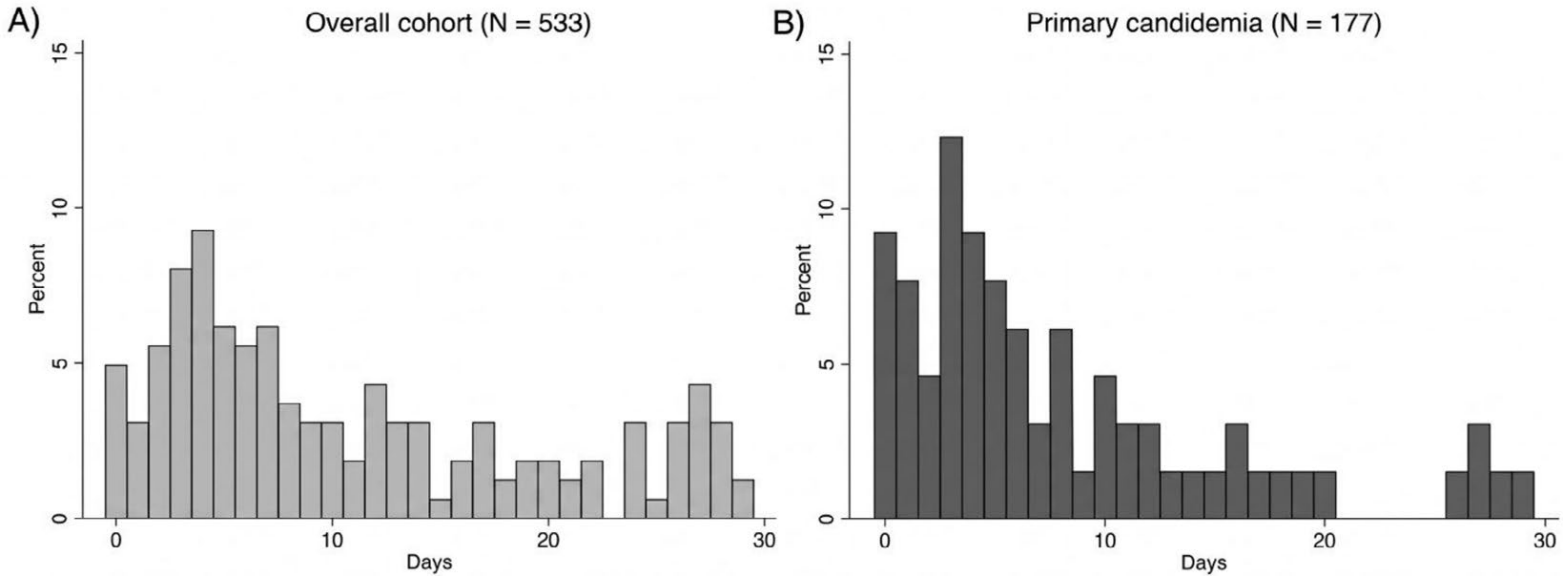
Daily mortality from the day of blood culture collection and during the first 30 days (A) in the overall cohort and (B) among the primary candidemia episodes.

Table 2 presents the factors associated with 7‐day mortality. Septic shock (OR 3.6, 95% CI: 2.1–6.3; *p* < 0.001) and primary candidemia (OR 2.2, 95% CI: 1.2–3.8; *p* = 0.011) were independently associated with increased 7‐day mortality.

**TABLE 2:** Predictors of 7‐day mortality in the study cohort.

Parameter	7‐day mortality (*N* = 88)	Alive (*N* = 445)	*p*	Multivariate OR (95% CI)	*p*
Sex (male)	54 (61.4)	289 (64.9)	0.522		
Age ≥ 65 years	45 (51.1)	235 (52.8)	0.774		
Septic shock	50 (56.8)	111 (24.9)	< 0.001	3.6 (2.0–6.3)	< 0.001
Mechanical ventilation	41 (46.6)	126 (28.3)	0.001	1.0 (0.6–1.8)	0.883
Underlying conditions					
Solid cancer	20 (22.7)	106 (23.8)	0.826		
Haematological malignancy	18 (20.5)	65 (14.6)	0.169		
Diabetes mellitus	23 (26.1)	106 (23.8)	0.643		
Chronic kidney failure	20 (22.7)	60 (13.5)	0.028	1.7 (0.9–3.1)	0.101
Liver disease	20 (22.7)	70 (15.7)	0.112		
HIV infection	4 (4.6)	14 (3.2)	0.509		
Solid organ transplant recipient	8 (9.1)	23 (5.2)	0.156		
Other disorders requiring or causing immunosuppression	8 (9.1)	24 (5.4)	0.187		
Haematopoietic stem cell transplantation	4 (4.6)	22 (4.9)	0.874		
Cardiovascular disease	28 (31.8)	155 (34.8)	0.587		
Risk factors for candidemia					
Neutropenia, (< 500 cells/μL)	11 (12.5)	29 (6.5)	0.056		
Major surgery within the previous month	30 (34.1)	143 (32.1)	0.720		
CVC^a^	65 (77.4)	332 (80.8)	0.477		
Parenteral nutrition	33 (37.5)	115 (25.8)	0.027	1.6 (1.0–2.7)	0.072
*Candida* species					
*Candida albicans*	29 (32.9)	144 (32.4)	0.913		
*Candida parapsilosis*	16 (18.2)	112 (25.2)	0.163		
*Nakaseomyces glabratus* ( *C. glabrata* )	26 (29.5)	111 (24.9)	0.367		
*Candida tropicalis*	7 (8.0)	45 (10.1)	0.534		
*Pichia kudriavzevii* ( *C. krusei* )	4 (4.6)	22 (4.9)	0.874		
Other *Candida* spp.	6 (6.8)	11 (2.5)	0.042	2.3 (0.8–6.7)	0.146
Breakthrough candidemia	15 (17.1)	69 (15.5)	0.717		
Source of candidemia					
Catheter‐related source	17 (19.3)	177 (39.8)	< 0.001	0.6 (0.3–1.3)	0.196
Primary candidemia	44 (50.0)	133 (29.9)	< 0.001	2.2 (1.2–3.8)	0.011
Abdominal source	14 (15.9)	66 (14.8)	0.796		
Urinary source	10 (11.4)	61 (13.7)	0.555		
Other source	3 (3.4)	8 (1.8)	0.340		
Early source control	29 (32.9)	147 (33.0)	0.988		
Initial Fluconazole treatment vs. echinocandin^b^	13 (20.6)	137 (31.0)	0.095		

Abbreviations: CI, confidence interval; CVC, catheter venous central; OR, odds ratio.

^a^ Among patients with a catheter in place at the time of candidemia diagnosis (*N* = 495, 84 died within 7 days and 411 survived).

^b^ Among patients who received antifungal treatment (*N* = 505, 63 died within 7 days and 442 survived).

Figure 1B depicts mortality over time among cases with primary candidemia. Given the high rate of 7‐day mortality among cases with primary candidemia (60% of them versus 41.2% in other sources, *p* < 0.001), the factors associated with the primary source were investigated. Male sex (OR 1.7, 95% CI 1.1–2.5; *p* = 0.016) and *N. glabratus* infection (OR 2.1, 95% CI: 1.3–3.3; *p* = 0.001) were independently associated with a primary source (Table 3).

**TABLE 3:** Predictors of primary candidemia in the study cohort.

Parameter	Primary candidemia (*N* = 177)	Other sources (*N* = 356)	*p*	Multivariate OR (95% CI)	*p*
Sex (male)	126 (71.2)	217 (61.0)	0.021	1.7 (1.1–2.5)	0.016
Age ≥ 65 years	78 (44.1)	202 (56.7)	0.006	0.7 (0.5–1.1)	0.097
Septic shock	52 (29.4)	109 (30.6)	0.769		
Neutropenia (< 500 cells/μL)	22 (12.4)	18 (5.1)	0.003	1.9 (0.9–4.2)	0.095
Parenteral nutrition	44 (24.9)	104 (29.2)	0.291		
Major surgery within the previous month	47 (26.6)	126 (35.4)	0.041		
Underlying conditions					
Solid cancer	35 (19.8)	91 (25.6)	0.140		
Haematological malignancy	38 (21.5)	45 (12.6)	0.009	1.3 (0.8–2.4)	0.313
Diabetes mellitus	39 (22.0)	90 (25.3)	0.410		
Chronic kidney failure	24 (13.6)	56 (15.7)	0.509		
Liver disease	37 (20.9)	53 (14.9)	0.082		
HIV infection	10 (5.7)	8 (2.3)	0.048	2.0 (0.7–5.4)	0.178
Solid organ transplant recipient	12 (6.8)	19 (5.3)	0.504		
Other disorders requiring or causing immunosuppression	10 (5.7)	22 (6.2)	0.808		
Haematopoietic stem cell transplantation	10 (5.7)	16 (4.5)	0.561		
Cardiovascular disease	51 (28.8)	132 (37.1)	0.059	0.8 (0.5–1.2)	0.336
*Candida* species					
*Candida albicans*	47 (26.6)	126 (35.4)	0.041	0.9 (0.6–1.4)	0.640
*Candida parapsilosis*	35 (19.8)	93 (26.1)	0.107		
*Nakaseomyces glabratus* ( *C. glabrata* )	63 (35.6)	74 (20.8)	< 0.001	2.1 (1.3–3.3)	0.001
*Candida tropicalis*	14 (7.9)	38 (10.7)	0.561		
*Pichia kudriavzevii* ( *C. krusei* )	10 (5.7)	16 (4.5)	0.313		
Other *Candida* spp.	6 (3.4)	8 (2.3)	0.440		
Breakthrough candidemia	21 (11.9)	63 (17.7)	0.084		

Abbreviations: CI, confidence interval; OR, odds ratio.

The 30‐day mortality differed according to the infection source. The lowest mortality was observed in catheter‐related candidemia (24.2%) and urinary source (26.8%), whereas markedly higher rates were seen in other sources (45.5%), abdominal source (41.3%), and primary candidemia (37.9%).

We explored factors in the overall cohort associated with 30‐day mortality. Septic shock (OR 3.1, IC95%: 1.9–5.1; *p* < 0.001), neutropenia (OR 2.5, IC95%: 1.0–5.8; *p* = 0.043), liver disease (OR 1.8, IC95%: 1.0–3.0; *p* = 0.043), and haematopoietic stem cell transplantation (OR 2.9, IC95%: 1.1–7.6; *p* = 0.034) were independently associated with increased 30‐day mortality (Table S1).

## 4 Discussion

Our study focused on the prevalence and clinical characteristics of primary candidemia and demonstrated that this source accounted for approximately one‐third of all candidemia episodes and was associated with substantial 7‐day mortality, with 60% of deaths occurring within the first 7 days after diagnosis. Considering that *Candida* spp. typically require more than 24 h to grow in blood cultures, initiation of appropriate antifungal therapy is often delayed, especially since only 34.3% of cases received active empirical antifungal treatment within the first 24 h. These findings highlight the need for faster diagnostic strategies; however, until these become widely available, early empirical antifungal treatment in selected high‐risk patients remains essential to improve short‐term outcomes.

The association observed between primary source and 7‐day mortality may reflect the early clinical course of candidemia, when the absence of an identifiable source may hinder the implementation of targeted source‐control measures, particularly in patients presenting with severe clinical conditions such as septic shock. In contrast, beyond the initial presentation (septic shock), 30‐day mortality appeared to be more strongly influenced by host‐related factors, particularly liver disease, neutropenia, and haematopoietic stem cell transplantation.

*N. glabratus* was the predominant species, accounting for 35.6% of primary candidemia episodes. Despite exhibiting fewer classical virulence factors than *C. albicans* , such as the lack of hyphal formation and lower tissue invasiveness, *N. glabratus* is associated with high rates of azole resistance and an enhanced capacity for host persistence, partly due to its ability to evade or attenuate host immune responses [13, 14]. *N. glabratus* is an opportunistic yeast particularly adapted to colonise the gastrointestinal tract, partly due to its ability to grow under anaerobic conditions [15]. Patients exposed to broad‐spectrum antibiotics, including critically ill patients, neutropenic individuals, and complex surgical patients, frequently develop intestinal dysbiosis and mucosal inflammation, conditions that may preferentially promote expansion of *N. glabratus* over other *Candida* species [16]. Taken together, our findings support a potential role for gastrointestinal translocation in the pathogenesis of episodes classified as primary candidemia, particularly those caused by *N. glabratus* .

Several factors may explain the poorer outcomes observed in these patients. First, unlike catheter‐related candidemia or abdominal collections, gastrointestinal translocation represents a source that cannot be directly controlled. Second, *N. glabratus* is associated with longer time‐to‐positivity values, frequently ≥ 48 h [17], potentially delaying microbiological confirmation and adequate antifungal adjustment. Finally, although echinocandins remain first‐line therapy, their activity against *N. glabratus* is generally lower than against other *Candida* species, particularly in isolates with elevated MICs or emerging resistance mechanisms [18]. Together, these factors may contribute to the excess mortality observed in our cohort.

These findings have potential implications for prevention and early diagnosis. Future studies should evaluate the individual positive and negative predictive values of rectal colonisation by *Candida* spp. in ICU patients for predicting subsequent candidemia, similarly to approaches proposed for multidrug‐resistant Gram‐negative bacteria [19]. Although the Candida Score already incorporates multifocal colonisation as a risk factor [20], the specific contribution of rectal colonisation by *N. glabratus* and *C. albicans* warrants further investigation, particularly because these species accounted for more than 60% of primary candidemia episodes in our cohort and are the dominant gut‐colonizing species. In addition, our findings may support further investigation into whether gut decolonisation strategies could have a potential role in preventing primary candidemia among patients with high‐density intestinal *Candida* colonisation.

Several limitations should be acknowledged. First, this was a retrospective single‐center study and is therefore subject to inherent biases. Second, some episodes may have been misclassified as primary candidemia due to incomplete investigation of the infectious focus or because early mortality precluded additional diagnostic procedures. Nevertheless, all episodes were systematically reviewed by infectious disease specialists; however, the retrospective design and the absence of a predefined standardised diagnostic work‐up prevent us from completely excluding this possibility. Third, rectal screening for *Candida* spp. is not routinely performed at our institution, limiting our ability to evaluate the relationship between colonisation and subsequent development of primary candidemia. Finally, we could not assess the impact of antifungal susceptibility because susceptibility data were not available throughout the entire study period.

## 5 Conclusion

Primary candidemia accounts for a substantial proportion of candidemia episodes and is associated with high 7‐day mortality. The predominance of *C. albicans* and *N. glabratus* , frequent gut colonisers, supports gastrointestinal translocation. Delayed microbiological diagnosis, particularly for *N. glabratus* , may contribute to poor outcomes, underscoring the importance of appropriate empirical antifungal therapy in high‐risk patients and the development of rapid diagnostic tools.

## Author Contributions

**Marta Albanell‐Fernández:** conceptualization, writing – original draft, writing – review and editing, methodology, data curation, formal analysis. **Andrea Vergara:** writing – review and editing. **Marta Bodro:** writing – review and editing. **Josep Mensa:** conceptualization, writing – review and editing. **Montse Tuset:** writing – review and editing. **Sabina Herrera:** writing – review and editing. **Álex Soriano:** conceptualization, supervision, writing – review and editing, methodology, formal analysis.

## Funding

The authors have nothing to report.

## Ethics Statement

The study was approved by the Ethics Committee of Hospital Clinic of Barcelona, Spain (HCB/2025/0796). The need for informed consent was waived due to the noninterventional, retrospective nature of the study.

## Conflicts of Interest

Álex Soriano has received honoraria for talks on behalf of Merck Sharp and Dohme, Pfizer, Novartis, Angelini, Menarini, Mundipharma, and Gilead Sciences, as well as grant support from Pfizer and Gilead Sciences. The rest of the authors declare no conflicts of interest.

## Supporting information


**Table S1:** Predictors of 30‐day mortality in the study cohort.

## Data Availability

The authors will make the data available upon reasonable request.
