# Supplementary material for: Unmasking Primary Candidemia: Clinical Features and Implications for Management
Source: Mycoses. 2026 Oct 23;69(10):e70228. doi: 10.1111/myc.70228 (PMC13691514; doi:10.1111/myc.70228)
Supplement: Supplementary file 1 [file MYC-69-e70228-s001.docx]

**SUPPLEMENTARY MATERIAL**

**Table S1.** Predictors of 30-day mortality in the study cohort.

| **Parameter** | **30-day mortality (N = 171)** | **Alive**  **(N = 362)** | ***P-value*** | **Multivariate OR**  **(95% CI)** | ***P-value*** |
| --- | --- | --- | --- | --- | --- |
| Sex (male) | 105 (61.4) | 238 (65.8) | 0.329 |  |  |
| Age ≥65 years | 94 (55.0) | 186 (51.4) | 0.439 |  |  |
| Septic shock | 85 (49.7) | 76 (21.0) | <0.001 | 3.1 (1.9–5.1) | <0.001 |
| Mechanical ventilation | 72 (42.1) | 95 (26.2) | <0.001 | 1.3 (0.8–2.1) | 0.378 |
| **Underlying conditions** | | | | | |
| Solid cancer | 43 (25.2) | 83 (22.9) | 0.574 |  |  |
| Hematological malignancy | 35 (20.5) | 48 (13.3) | 0.033 | 1.1 (0.6–2.3) | 0.724 |
| Diabetes mellitus | 44 (25.7) | 85 (23.5) | 0.571 |  |  |
| Chronic kidney failure | 30 (17.5) | 50 (13.8) | 0.265 |  |  |
| Liver disease | 38 (22.2) | 52 (14.4) | 0.025 | 1.8 (1.0–3.0) | 0.043 |
| HIV infection | 6 (3.5) | 12 (3.3) | 0.908 |  |  |
| Solid organ transplant recipient | 11 (6.4) | 20 (5.5) | 0.676 |  |  |
| Other disorders requiring or causing immunosuppression | 14 (8.2) | 18 (5.0) | 0.149 |  |  |
| Hematopoietic stem cell transplantation | 14 (8.2) | 12 (3.3) | 0.018 | 2.9 (1.1–7.6) | 0.034 |
| Cardiovascular disease | 57 (33.3) | 126 (34.8) | 0.738 |  |  |
| **Risk factors for candidemia** | | | | | |
| Neutropenia, (<500 cells/µL) | 21 (12.3) | 19 (5.3) | 0.005 | 2.5 (1.0–5.8) | 0.043 |
| Major surgery within the previous month | 51 (29.8) | 122 (33.7) | 0.372 |  |  |
| CVC^a^ | 132 (80.5) | 265 (80.1) | 0.911 |  |  |
| Parenteral nutrition | 58 (33.9) | 90 (24.9) | 0.030 | 1.4 (0.9–2.2) | 0.193 |
| ***Candida* species** | | | | | |
| *Candida albicans* | 55 (32.2) | 118 (32.6) | 0.921 |  |  |
| *Candida parapsilosis* | 30 (17.5) | 98 (27.1) | 0.034 | 0.8 (0.5–1.4) | 0.431 |
| *Nakaseomyces glabratus* (*C. glabrata*) | 54 (31.6) | 83 (22.9) | 0.017 | 1.1 (0.7–1.9) | 0.668 |
| *Candida tropicalis* | 18 (10.5) | 34 (9.4) | 0.681 |  |  |
| *Pichia kudriavzevii* (*C. kruse*i) | 8 (4.7) | 18 (5.0) | 0.883 |  |  |
| Other *Candida* spp. | 6 (3.5) | 9 (2.5) | 0.768 |  |  |
| Breakthrough candidemia | 29 (17.0) | 55 (15.2) | 0.602 |  |  |
| **Source of candidemia** | | | | | |
| Catheter-related source | 47 (27.5) | 147 (40.6) | 0.003 | 0.8 (0.5–1.4) | 0.404 |
| Primary candidemia | 67 (39.2) | 110 (30.4) | 0.046 | 0.9 (0.5–1.6) | 0.756 |
| Abdominal source | 33 (19.3) | 47 (13.0) | 0.061 |  |  |
| Urinary source | 19 (11.1) | 52 (14.4) | 0.303 |  |  |
| Other source | 5 (2.9) | 6 (1.7) | 0.343 |  |  |
| Early source control | 53 (31.0) | 123 (34.0) | 0.494 |  |  |
| Initial Fluconazole treatment vs. echinocandin^b^ | 33 (22.8) | 117 (32.5) | 0.031 | 0.8 (0.5–1.3) | 0.348 |

Abbreviations: CI: Confidence interval; CVC: Catheter venous central; OR: Odds ratio.

*^a^*Among patients with a catheter in place at the time of candidemia diagnosis (N = 495, 164 died and 331 survived).

^b^Among patients with a catheter in place at the time of candidemia diagnosis (N = 505, 145 died and 360 survived).
